# S100A9 and ORM1 serve as predictors of therapeutic response and prognostic factors in advanced extranodal NK/T cell lymphoma patients treated with pegaspargase/gemcitabine

**DOI:** 10.1038/srep23695

**Published:** 2016-03-29

**Authors:** Zhiyuan Zhou, Zhaoming Li, Zhenchang Sun, Xudong Zhang, Lisha Lu, Yingjun Wang, Mingzhi Zhang

**Affiliations:** 1 Department of Oncology, The First Affiliated Hospital of Zhengzhou University, No.1 Jianshe East Road, Zhengzhou, 450000, China; 2 Institute of Clinical Medicine, The First Affiliated Hospital of Zhengzhou University, No. 1 Jianshe East Road, Zhengzhou, 450000, China

## Abstract

Pegaspargase combined with gemcitabine have greatly improved the outcomes of advanced extranodal NK/T cell lymphoma (ENKL). However, patients frequently undergo recurrent disease due to chemoresistance, and few predictive parameters are available. The present study explored potential biomarkers to predict the therapeutic response of advanced ENKL treated with pegaspargase/gemcitabine and evaluate the prognostic significance. Through serum proteomic analysis, we identified 61 upregulated and 22 downregulated proteins in nonresponders compared with responders. We further validated that patients with unfavourable treatment outcomes displayed higher levels of S100A9 and ORM1 via enzyme-linked immunosorbent assay (ELISA). Moreover, the sensitivity and specificity for detecting refractory patients were 81.5% and 71.4% for S100A9 > 62.0 ng/ml, 85.2% and 77.1% for ORM1 > 1436 ug/ml, 100% and 57.1% for S100A9 combined with ORM1. Furthermore, in multivariate analysis elevated levels of S100A9 were associated with poor 2-year OS (40.2% vs. 76.6%, RR = 2.92, p = 0.005) and 2-year PFS (33.1% vs. 61.1%, RR = 2.61 p = 0.011). High ORM1 also predicted inferior 2-year OS (38.7% vs.76.1, RR = 2.46, p = 0.023) and 2-year PFS (18.4% vs. 73.2%, RR = 2.86, p = 0.009). Our results indicated that S100A9 and ORM1 could serve as reliable predictors of therapeutic response and independent prognostic factors of survival in advanced ENKL patients treated with pegaspargase/gemcitabine.

Extranodal NK/T cell lymphoma (ENKL) represents a highly aggressive lymphoid malignancy characterized by dismal survival. As an Epstein-Barr Virus (EBV)-associated disease, populations from Asian countries and South America are more frequently affected^1^. Gene expression profiling (GEP) analysis suggested that activation of JAK-STAT, NF-κB and MAPK signaling pathways contributes to the pathogenesis of ENKL^2^. Due to the rarity of this disease, standard therapies for ENKL have not been established. Most localized patients could achieve remission after receiving radiotherapy and chemotherapy^3^. As to advanced ENKL patients, high-dose chemotherapy containing pegaspargase, L-asparaginase, gemcitabine are recommended. The SMILE (methotrexate, ifosfamide, L-asparaginase, dexamethasone and etoposide) regimen and DDGP (gemcitabine, pegaspargase, cisplatin, and dexamethasone) regimen have showed promising efficacy^4^,^5^. Unfortunately, some patients still experience refractory disease and undergo disease progression. Currently, few parameters except EBV-DNA copy numbers are reported to be correlated with therapeutic response in ENKL^6^,^7^. For those nonresponders and high risk patients, modified therapy strategy would prolong their survival. Investigation of reliable biomarkers for predicting treatment response are urgently needed.

Proteomic technology has emerged as a valuable tool to screen alterations in protein profilings of serum^8^. In cancer development, crucial proteins from cancer cells and tumor microenvironment are secreted into serum. Serum samples provide us a great much information. Biochemical analysis of serum may serve as a noninvasive method for cancer diagnosis and prognosis. Through quantitative proteomic analysis, specific protein signatures for meningiomas with different grades were identified^9^. We previously employed proteomic techniques and identified a protein that could distinguish healthy individuals and lymphoma patients^10^.

Here, we utilized proteomic approach to identify differentially expressed proteins between chemosensitive- and chemoresistant- advanced ENKL patients. Nonresponders presented higher levels of S100A9 and ORM1 than responders. Elevated levels of S100A9 and ORM1 were associated with shorter PFS and OS. Our results indicated that serum S100A9 and ORM1 could serve as powerful predictors of chemoresponsiveness and prognostic factors of survival in advanced ENKL treated with pegaspargase/gemcitabine.

## Materials and Methods

### Patients

For isobaric tags for relative and absolute quantitation (iTRAQ) and LC-MS/MS (liquid chromatography tandem mass spectrometry) analysis, pretreatment blood samples from 32 advanced ENKL patients were collected. For validation set, another cohorts of 62 advanced ENKL patients were enrolled. Blood samples were placed at room temperature for 1 hour to allow coagulation, and then centrifuged at 3500 rpm for 10 min at 4 °C. Serums were then aliquoted and stored at −80 °C until further experiment. All patients received DDGP (gemcitabine, pegaspargase, cisplatin, and dexamethasone) chemotherapy regimen. Assessment of treatment response was based on Revised Cheson’s standard response criteria. Complete remission (CR), partial response (PR), stable disease (SD), progressive disease (PD) were defined as previously described^5^. According to response and PFS, patients were divided into responders and nonresponders. Responders were those who achieved CR or PR and did not undergo disease progression within 6 months after therapy. Nonresponders were defined as those who achieved SD/PD after initial therapy or those who obtained temporary remission but relapsed within 6 months.

This study was approved by the ethics committee of the First Affiliated Hospital of Zhengzhou University. All experiments were performed in accordance with approved guidelines and regulations. All patients signed an informed consent when enrolled.

### iTRAQ labeling and strong cation exchange (SCX) chromatography

Equal amounts of serum from each sample were acquired and pooled. Multiple Affinity Removal LC Column-Human 14 (Agilent) was employed to remove high abundance serum proteins. Bradford assay was performed to determine the concentrations of proteins. Then, proteins were digested into peptides with trypsin and desalted with C18-SD Extraction Disk Cartridge (66872-U sigma). Peptides were labeled with reagents following the iTRAQ kit (AB SCIEX) instructions and then mixed. For LC-MS/MS analysis, the complexity of peptides sample was firstly reduced utilizing strong cation exchange (SCX) chromatography. The labeled peptides were separated using a Polysulfoethyl column (4.6 × 100 mm, 5 um, 200 Å, PolyLCInc, Maryland, USA) on the AKTA Purifier 100 system (GE Healthcare). The peptide fractions were collected at an interval of one minute and grouped into 12 components according to SCX chromatograms. Components were lyophilized in a vacuum concentrator and desalted using C18 columns.

### LC-MS/MS

Each component was further analyzed using a Q Exactive mass spectrometer (Thermo Finnigan) connected to the Easy nano Liquid Chromatography system. Briefly, dried fraction was resuspended in buffer A (0.1% formic acid) and packed with C18-reversed phase column (2 cm × 100 μm, 5 μm, Thermo Scientific). Peptides were eluted onto an analytical C18 column (75 um × 100 mm, 3 μm, Thermo Scientific) with a gradients of buffer B (84% acetonitrile and 0.1% Formic acid) at a flow rate of 300 nl/min over 60 min. The mass spectrometer was performed at a data-dependent mode. In a full MS scan range of m/z 300–1800, the ten most abundant precursor ions were choosed for high-energy collisional dissociation (HCD) fragmentation. Survey scans were acquired at a resolution of 70,000 at m/z 200 and resolution for HCD spectra was set to 17,500 at m/z 200.

### Protein identification and bioinformatic analysis

For peptide and protein identification, tandem mass spectra were searched against a nonredundant International Protein Index arabidopsis sequence database v3.85 using Mascot2.2 (Matrix Science, London, UK; version 2.2) and proteome Discoverer1.4 (Thermo). The MASCOT search results for each SCX elution were further processed using the ProteomicsTools (version 3.05). All reported data were based on 99% confidence for protein identification as determined by false discovery rate (FDR) ≤1%. Quantification of proteins were performed according to intensity of report ions in each identified peptide. The differentially expressed proteins were searched against SwissProt database to find homologue sequences followed by gene ontology (GO) annotation and mapping. These proteins were also blasted against KEGG GENES and mapped to pathways in KEGG.

### Enzyme-linked immunosorbent assay (ELISA)

Among the differentially expressed proteins between responders and nonresponders, S100A9 and ORM1 were selected as candidate biomarkers for further validation in another cohorts of 62 advanced ENKL patients. Serum levels of S100A9 and ORM1 were measured using commercially available ELISA Kit (Uscn Life Science, Wuhan, China) according to the manufacturer’s instructions.

### Statistical analysis

The median levels of S100A9 and ORM1 from responders and nonresponders were compared by Mann Whitney tests. Receiver-operating-characteristics (ROC) analysis was employed to determine the cutoff concentration of S100A9 and ORM1 and evaluate the predictive accuracy. Overall survival (OS) was calculated from the date of the initial therapy until the date of death or the last follow-up. PFS was calculated from the date of the initial therapy until the date of disease progression or death from any cause. Survival analysis was performed using the Kaplan-Meier method and the log-rank test. Multivariate analysis using Cox regression model was conducted using SPSS 17.0.

## Results

### Identification of differentially expressed proteins in serums between responders and nonresponders

**Figure 1:**
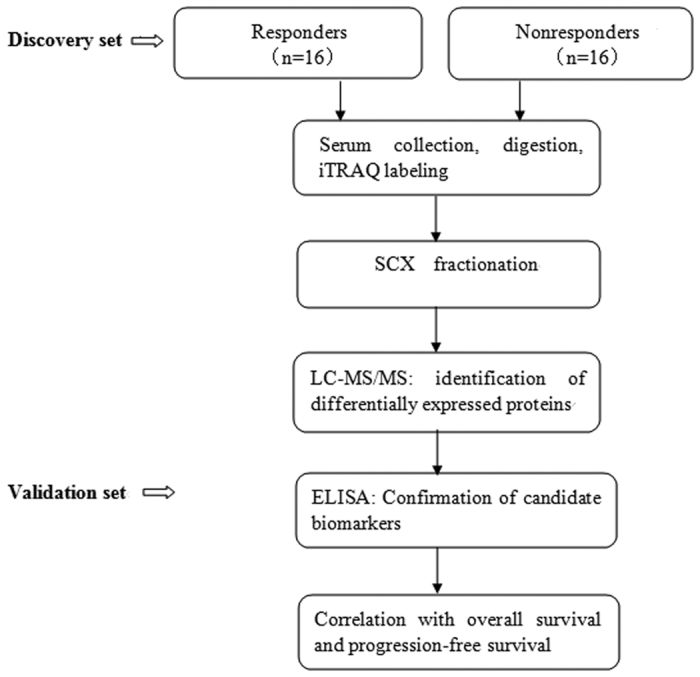
The workflow of how to discover and validate biomarkers.

To explore potential biomarkers associated with treatment response in advanced ENKL patients, pretreatment serums from 16 responders and 16 nonresponders were collected. The clinicopathologic characteristics of the two groups were matched. The workflow of the discovery phase was shown in Fig. 1. After digested with trypsin and labeled with iTRAQ regents, the peptides were pooled. SCX chromatography was performed to reduce sample complexity. The fractions were then analyzed by a Q Exactive mass spectrometer connected to the Easy nano Liquid Chromatography. Comparative proteomic analysis identified 22 downregulated and 61 upregulated proteins with local false discovery rate (FDR) <5% in nonresponders (Supplementary 1). The upregulated proteins in nonresponders included S100A9, ORM1, alpha-1-antitrypsin, 14-3-3 protein zeta/delta. The downregulated proteins included platelet factor 4, vitamin D-binding protein, thrombospondin-1 and gelsolin. These differentially expressed proteins were mainly involved in the process of inflammatory/acute phase response, cell cycle and DNA damge (Supplementary 2). KEGG pathway analysis indicated that the dysregulated proteins were enriched in chemokine signaling pathway, hippo signaling pathway and PI3K-Akt signaling pathway.

### Validation of the candidate biomarkers

**Figure 2:**
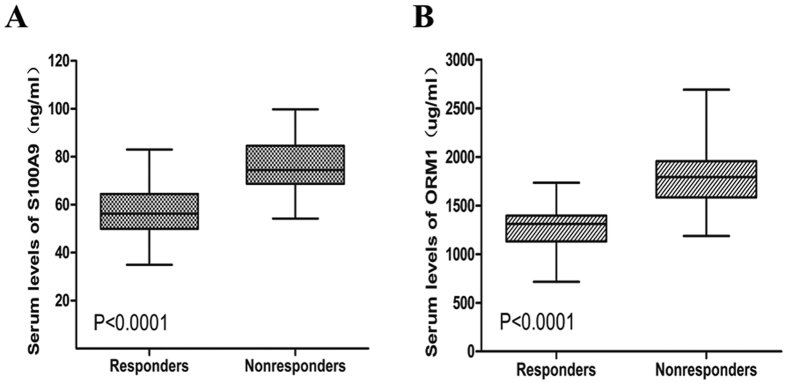
Levels of S100A9 and ORM1 in serums from responders and nonresponders. (**A**) Nonresponders displayed higher levels of S100A9 (P < 0.0001). (**B**) Nonresponders displayed higher levels of ORM1 (P < 0.0001).

From the above datas, we found that serum levels of S100A9 and ORM1 were significantly elevated in patients who obtained unfavourable outcomes. Since inflammation and EBV infection were deeply involved in the development of ENKL, we assumed that inflammatory factors such as S100A9 and ORM1 may be associated with treatment response and the prognosis of ENKL. ELISA assay was performed to validate S100A9 and ORM1 in another cohort of 62 patients. The baseline characteristics of patients were shown in Table 1. Serum levels of S100A9 and ORM1 were measured respectively. As shown in Fig. 2A, serum levels of S100A9 were significantly higher in nonresponders than that in responders. In Fig. 2B, nonresponders also presented higher levels of ORM1 than responders. The absolute quantification of S100A9 and ORM1 by ELISA allowed us to determine a threshold to differentiate low-S100A9 group and high-S100A9 group, low-ORM1 group and high-ORM1 group. ROC curve analysis indicated that serum levels of S100A9 at the concentration of 62.0 ng/ml possessed a sensitivity of 81.5% and a specificity of 71.4%. The cutoff point for ORM1 was 1436 ug/ml, with a sensitivity of 85.2% and a specificity of 77.1%. When S100A9 in combined with ORM1, the sensitivity increased to 100% (Table 2).

### Correlation of S100A9 and ORM1 with overall survival and progression-free survival

**Figure 3:**
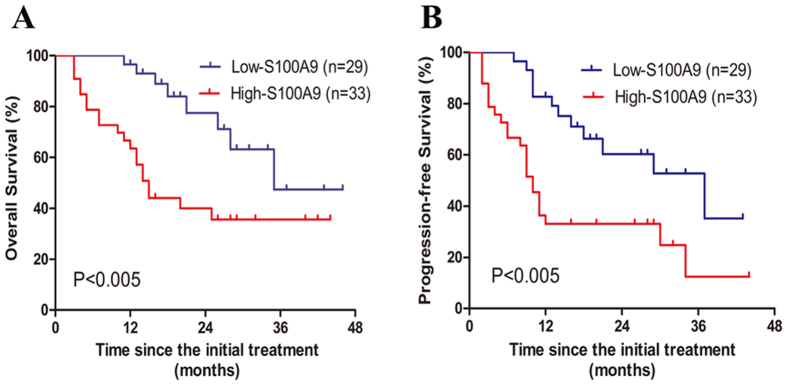
Survival of low-S100A9 group and high-S100A9 group. **(A**) High levels of S100A9 in serum were associated with inferior overall survival (P = 0.0036). (**B**) High levels of S100A9 in serum were associated with inferior progression-free survival (P = 0.0019).

**Figure 4:**
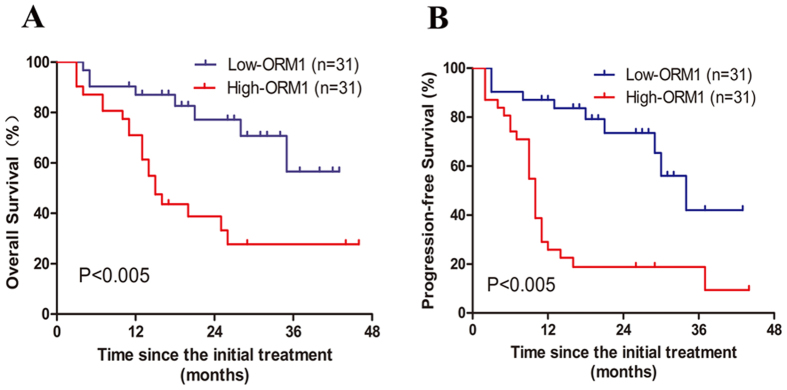
Survival of low-ORM1 group and high-ORM1 group. **(A**) High levels of ORM1 in serum were associated with inferior overall survival (P = 0.0012). (**B**) High levels of ORM1 in serum were associated with inferior progression-free survival (P < 0.0001).

**Figure 5:**
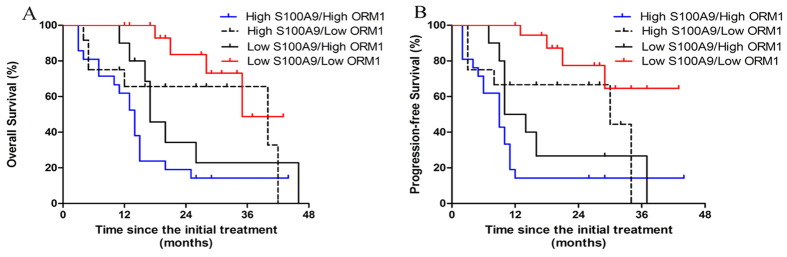
The significance of combination of S100A9 and ORM1 in survival. **(A**) Patients with high S100A9/high ORM1 had poor 2-year overall survival (p < 0.005). (**B**) Patients with high S100A9/high ORM1 had poor 2-year progression-free survival (p < 0.005).

To address the clinical relevance, serum levels of S100A9 and ORM1 were correlated with OS and PFS. The median follow-up is 17 months (range, 3–46 months). As shown in Fig. 3A, the 2-year OS for low-S100A9 group and high-S100A9 group were 76.6% and 40.2% (P = 0.0036), respectively. High-S100A9 group also predicted poor 2-year PFS in comparision with low-S100A9 group (33.1% vs 61.1%, p = 0.0019) (Fig. 3B). In terms of ORM1, high levels of ORM1 were associated with inferior 2-year OS (38.7% vs.76.1, p = 0.0012) and 2-year PFS (18.4% vs. 73.2%, p < 0.0001) (Fig. 4A,B). Furthermore, multivariate analysis using Cox regression model was conducted to explore potential prognostic factors for advanced ENKL patients. S100A9 > 62 ng/ml (RR = 2.92, 95% CI: 1.37–6.22, P = 0.005) and ORM1 > 1436 ug/ml (RR = 2.46, 95% CI: 1.14–5.32, P = 0.023) were independent predictors of overall survival. Elevated S100A9 (RR = 2.46, 95% CI: 1.14–5.32, P = 0.023) and ORM1 (RR = 2.86, 95% CI = 1.30–6.27, P = 0.009) were also adverse factors for PFS (Table 3). As shown in Fig. 5A, patients with high S100A9/high ORM1 had poor 2-year OS than those with high S100A9/low ORM, low S100A9/high ORM1, low S100A9/low ORM1 (19.05% vs. 66.67%, 34.28, 83.57%, p < 0.005). Moreover, the 2-year PFS for patients with high S100A9/high ORM1 and high S100A9/low ORM, low S100A9/high ORM1, low S100A9/low ORM1 were 14.29% and 65.63%, 26.67%, 77.49%, respectively (P < 0.005) (Fig. 5B). These results showed that high S100A9/high ORM1 predicted poor prognosis in advanced ENKL patients.

## Discussion

Chemotherapy regimens containing pegaspargase, l-asparaginase, gemcitabine have shown remarkable efficacy in advanced ENKL. However, some patients still experience treatment failure and recurrent disease. Currently, few parameters are available to discriminate patients who would achieve unfavorable responses.

In the present study, we firstly conducted proteomic analysis to identify differentially expressed proteins in serum from responders and nonresponders. S100A9 and ORM1 were upregulated in nonresponders compared to responders. ELISA assay further confirmed that refractory patients displayed higher levels of S100A9 and ORM1. S100A9 in combined with ORM1 could effectively detect refractory patients with a sensitivity of 100%. Moreover, high-S100A9 and high-ORM1 were associated with inferior OS and PFS. Mutivariate analysis indicated that both S100A9 and ORM1 are independent prognostic factors of survival in advanced ENKL.

S100A9 is a calcium binding protein implicated in tumor growth. It interacts with toll-like receptor 4 (TLR4) or receptor for advanced glycation end products (RAGE) on the surface of cancer cells, which mediates activation of MAPK, NF-Kb, and AKT signalings^11^,^12^. Moreover, S100A9 can recruit myeloid derived suppressor cells (MDSC) in tumor sites and result in invasive phenotype of cancer^13^. Increased expression of S100A9 was observed in colitis-associated colon cancer, gastric cancer and lung cancer^14^. It binds to the RAGE or TLR on tumor cells, and thus activates oncogenic pathways and mediates inflammatory cascades^15^,^16^. *In vitro* studies indicated S100A9 at low concentration promoted the proliferation of hepatocellular carcinoma cells and metastasis of breast cancer^17^,^18^. Moreover, S100A9 can recuit MDSC in tumor sites, which suppresses T cell function and promote tumor metastasis. Growth of lymphoma tumor in S100A9-deficient mice was slower than that in wild-type mice, predominantly due to reduced recruitment of MDSCs^19^. Overexpression of S100A9 confers to chemoresistance. Advanced muscle invasive bladder cancer patients who displayed higher levels of S100A9 were insensitive to cisplatin-based chemotherapy and more frequently experienced disease progression^20^. For early-stage oral cancer, elevated S100A9 expression in tumor stroma increases macrophage recruitment and functions as an early recurrence marker^21^. In the current study, levels of S100A9 were elevated in chemoresistant ENKL patients.

ORM1, an acute phase protein, increases in serum as a response to inflammation. In addition, elevation of ORM1 were discovered in certain cancer patients^22^. Most cancer patients displayed increased levels of ORM1 in comparision with healthy controls. The study by Ganz PA and colleagues found that plasma levels of ORM1 were elevated in 89% of the solid tumor patients and 87% of the patients with hematologic neoplasms^22^. The prognostic values of ORM1 have also been demonstrated. In non-small cell lung cancer patients treated with docetaxel, the response rate for high baseline ORM1 group was 14%, while for low ORM1 group it was 44%. Patients with high ORM1 had a striking shorter survival than low-ORM1 patients^23^. Serum ORM1 also predicts the responses to neoadjuvant chemotherapy in advanced breast cancers^24^. Our results found that advanced ENKL patients who were sensitive to pegaspargase/gemcitabine treatment displayed lower ORM1 in serum. ENKL patients with low ORM1 had superior OS and PFS than high ORM1 patients.

Inflammation and EBV infection are deeply implicated in the pathogenesis of ENKL. However, the exact mechanisms underlying the role of EBV infection and inflammation in the development of ENKL remain largely unknown. The prognostic values of EBV-DNA and inflammatory factors in ENKL patients have been demonstrated previously. Ito Y and colleagues showed that pretreatment EBV-DNA copy number in whole blood was helpful to predict tumor response and survival^7^. Recent studies suggested that serum ferritin, interleukin-10 (IL-10) and c-reactive protein (CRP) could also serve as independent prognostic factors in ENKL^25^,^26^,^27^. Both S100A9 and ORM1 are inflammatory factors with multiple acyivities. Further studies would focus on how S100A9 and ORM1 affect the invasive type and drug sensitivity of ENKL cells.

In conclusion, we firstly identified S100A9 and ORM1 as novel serum biomarkers to predict the therapeutic response of advanced ENKL patients to pegaspargase/gemcitabine treatment. S100A9 and ORM1 could also serve as independent prognostic factors of survival in advanced ENKL.

## Additional Information

**How to cite this article**: Zhou, Z. *et al.* S100A9 and ORM1 serve as predictors of therapeutic response and prognostic factors in advanced extranodal NK/T cell lymphoma patients treated with pegaspargase/gemcitabine. *Sci. Rep.* **6**, 23695; doi: 10.1038/srep23695 (2016).

## Supplementary Material

Supplementary Information

## Figures and Tables

**Table 1:** Characteristics of ENKL patients (n = 62).

Characteristics	n		%
Sex			
Male	43		69.35
Female	19		30.65
Age(years)			
Median		41	
Range		14–76	
LDH			
Normal	27		43.55
Elevated	35		56.45
B symptom			
Negative	30		48.39
Positive	32		51.61
IPI			
≤2	33		53.23
>2	29		46.77
EBV-DNA			
<5000 copies/ml	30		48.39
≥5000 copies/ml	32		51.61
S100A9			
Low level (<62 ng/ml)	29		46.77
High level (≥62 ng/ml)	33		53.23
ORM1			
Low level (<1436 ug/ml)	31		50
High level (≥1436 ng/ml)	31		50
Response			
Responders	35		56.45
Nonresponders	27		43.55

Abbreviations: ENKL, extranodal NK/T cell lymphoma; LDH, lactate dehydrogenase; IPI, international prognostic index; EBV, Epstein-Barr Virus.

**Table 2:** The sensitivity and specificity of S100A9, ORM1, S100A9 in combined with ORM1 for detecting nonresponders.

	Nonresponders(n = 27)			Responders(n = 35)		
	positive (a)	negative (b)	sensitivity	positive (c)	negative (d)	specificity
S100A9	22	5	81.5%	25	10	71.4%
ORM1	23	4	85.2%	27	8	77.1%
S100A9 + ORM1	27	0	100%	20	15	57.1%

Sensitivity = a/a + b, Specificity = c/c + d.

**Table 3:** Univariate and multivariate analysis of OS and PFS in advanced ENKL patients.

Variables	OS		*P*		PFS	*P*
	Univariate analysis	Multivariate analysis		Univariate analysis	Multivariate analysis	
	*P*	RR(95% CI)		*P*	RR(95% CI)	
Age > 60	0.097			0.106		
Male	0.323			0.349		
LDH > 245U/L	0.028			0.043		
B symptom	0.100			0.164		
IPI ≥ 3	0.057			0.073		
EBV-DNA > 5000 copies/ml	0.0002	3.95(1.74–8.97)	0.001	<0.0001	3.91(1.76–8.68)	0.001
S100A9 > 62 ng/ml	0.0036	2.92(1.37–6.22)	0.005	0.0019	2.61(1.25–5.45)	0.011
ORM1 > 1436 ug/ml	0.0012	2.46(1.14–5.32)	0.023	<0.0001	2.86(1.30–6.27)	0.009

Abbreviations: LDH, lactate dehydrogenase; IPI, international prognostic index; EBV, Epstein-Barr Virus.
