# Supplementary material for: S100A9 and ORM1 serve as predictors of therapeutic response and prognostic factors in advanced extranodal NK/T cell lymphoma patients treated with pegaspargase/gemcitabine
Source: Sci Rep. 2016 Mar 29;6:23695. doi: 10.1038/srep23695 (PMC4810364; doi:10.1038/srep23695)
Supplement: Supplementary Information [file srep23695-s1.docx]

**S100A9 and ORM1** **serve as predictors of therapeutic response and prognostic factors in advanced extranodal NK/T cell lymphoma patients treated** **with** **pegaspargase/gemcitabine**

Zhiyuan Zhou^1,2^, Zhaoming Li^1^, Zhenchang Sun^1^, Xudong Zhang^1^, Lisha Lu^1,2^, Yingjun Wang^1,2^ & Mingzhi Zhang^1^

**Supplement 1. Mapping of dysregulated proteins to biological and cellular processes using gene ontology (GO) analysis.**

**
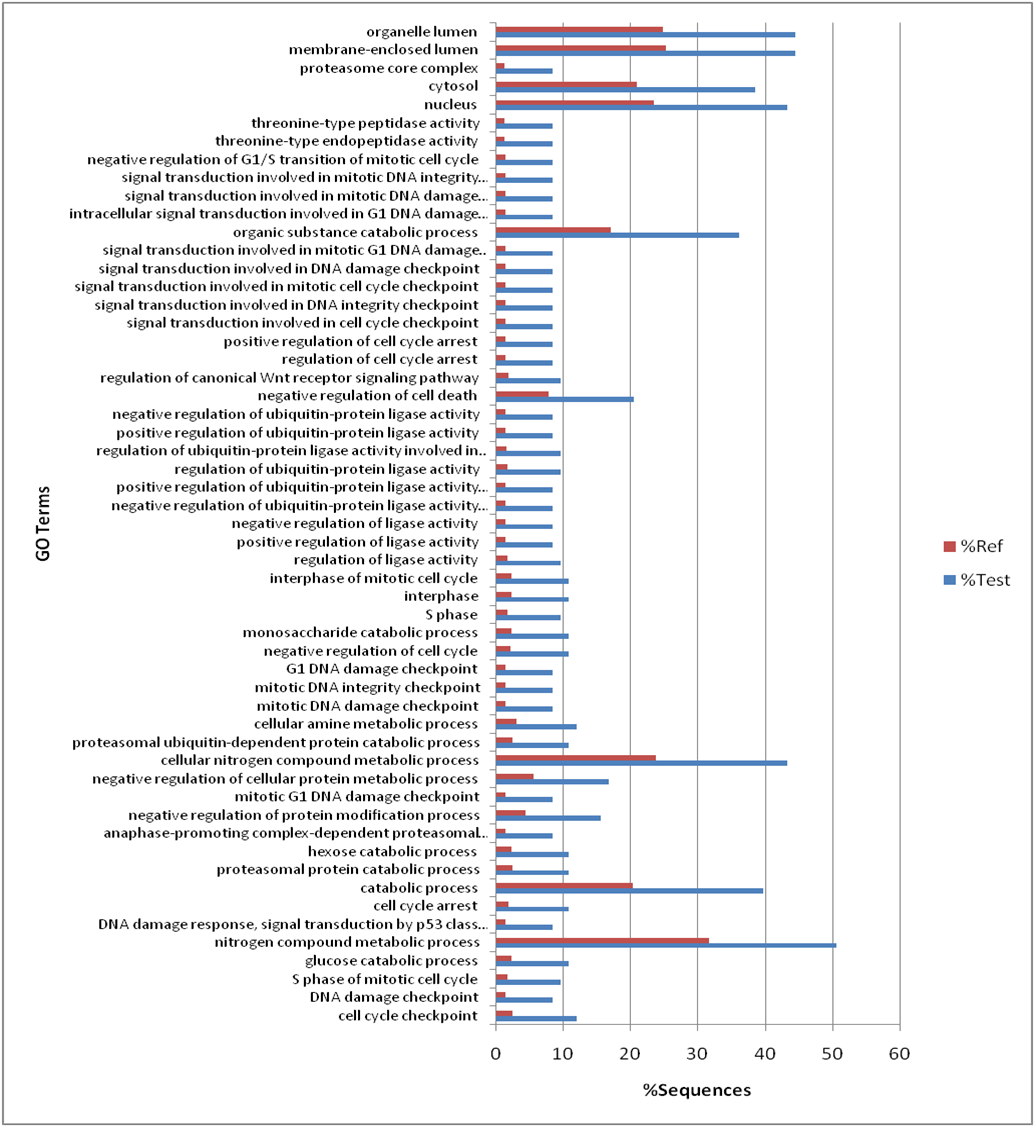
**

**Supplement 2. Mapping of dysregulated proteins to pathways using KEGG pathway analysis.**

**
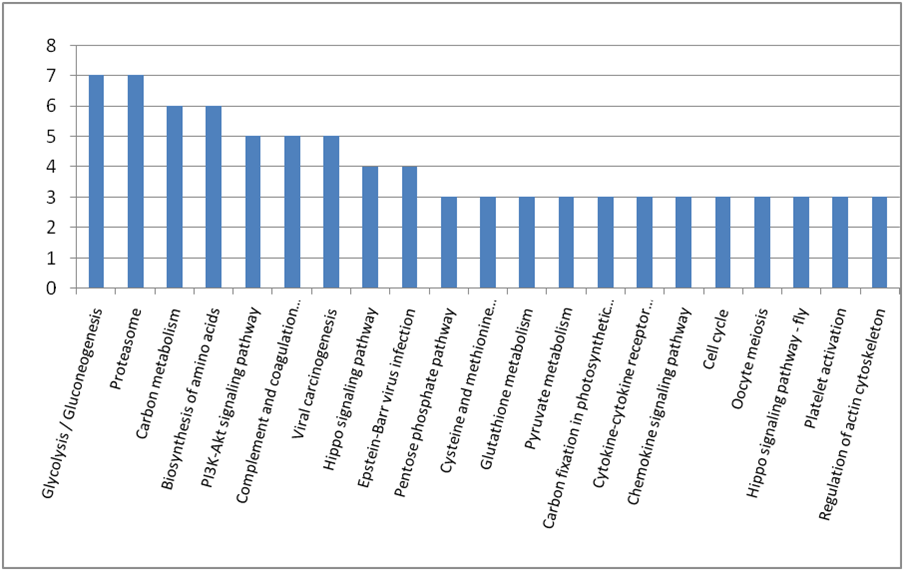
**
